# Human intravenous immunoglobulin provides protection against Aβ toxicity by multiple mechanisms in a mouse model of Alzheimer's disease

**DOI:** 10.1186/1742-2094-7-90

**Published:** 2010-12-07

**Authors:** Johanna Magga, Lakshman Puli, Rea Pihlaja, Katja Kanninen, Suvi Neulamaa, Tarja Malm, Wolfgang Härtig, Jens Grosche, Gundars Goldsteins, Heikki Tanila, Jari Koistinaho, Milla Koistinaho

**Affiliations:** 1 Department of Neurobiology, A. I. Virtanen Institute for Molecular Sciences, University of Eastern Finland, Kuopio, Finland; 2 Paul Flechsig Institute for Brain Research, University of Leipzig, Leipzig, Germany; 3 Department of Neurology, Kuopio University Hospital, Kuopio, Finland; 4 Department of Oncology, Kuopio University Hospital, Kuopio, Finland; 5 School of Pharmacy, University of Eastern Finland, Kuopio, Finland; 6 Medeia Therapeutics Ltd., Kuopio, Finland

## Abstract

**Background:**

Purified intravenous immunoglobulin (IVIG) obtained from the plasma of healthy humans is indicated for the treatment of primary immunodeficiency disorders associated with defects in humoral immunity. IVIG contains naturally occurring auto-antibodies, including antibodies (Abs) against β-amyloid (Aβ) peptides accumulating in the brains of Alzheimer's disease (AD) patients. IVIG has been shown to alleviate AD pathology when studied with mildly affected AD patients. Although its mechanisms-of-action have been broadly studied, it remains unresolved how IVIG affects the removal of natively formed brain Aβ deposits by primary astrocytes and microglia, two major cell types involved in the neuroinflammatory responses.

**Methods:**

We first determined the effect of IVIG on Aβ toxicity in primary neuronal cell culture. The mechanisms-of-action of IVIG in reduction of Aβ burden was analyzed with *ex vivo *assay. We studied whether IVIG solubilizes natively formed Aβ deposits from brain sections of APP/PS1 mice or promotes Aβ removal by primary glial cells. We determined the role of lysosomal degradation pathway and Aβ Abs in the IVIG-promoted reduction of Aβ. Finally, we studied the penetration of IVIG into the brain parenchyma and interaction with brain deposits of human Aβ in a mouse model of AD *in vivo*.

**Results:**

IVIG was protective against Aβ toxicity in a primary mouse hippocampal neuron culture. IVIG modestly inhibited the fibrillization of synthetic Aβ1-42 but did not solubilize natively formed brain Aβ deposits *ex vivo*. IVIG enhanced microglia-mediated Aβ clearance *ex vivo*, with a mechanism linked to Aβ Abs and lysosomal degradation. The IVIG-enhanced Aβ clearance appears specific for microglia since IVIG did not affect Aβ clearance by astrocytes. The cellular mechanisms of Aβ clearance we observed have potential relevance *in vivo *since after peripheral administration IVIG penetrated to mouse brain tissue reaching highest concentrations in the hippocampus and bound selectively to Aβ deposits in co-localization with microglia.

**Conclusions:**

Our results demonstrate that IVIG promotes recognition and removal of natively formed brain Aβ deposits by primary microglia involving natural Aβ Abs in IVIG. These findings may have therapeutic relevance *in vivo *as IVIG penetrates through the blood-brain barrier and specifically binds to Aβ deposits in brain parenchyma.

## Background

Deposition of Aβ peptides is the major hallmark of AD in addition to neurofibrillary tangles formed by hyperphosphorylated tau [1]. The Aβ deposits consist primarily of fibrillized Aβ1-40 and Aβ1-42 peptides, the latter being more prone to aggregation. The Aβ deposits containing Aβ peptide oligomers, diffuse Aβ deposits and aggregated fibrillar Aβ induce neurotoxicity and cognitive defects, as demonstrated *in vitro *and *in vivo *[1-4]. The Aβ neurotoxicity may be largely regulated by microglia, the surveillant cells of the CNS [5], which may possess double-faced actions of conducting both pro-inflammatory and anti-inflammatory effects [6-9].

The reduction of Aβ burden by passive immunization has been shown to alleviate neurodegeneration and cognitive defects in mouse models of AD [10-13]. There are numerous potential mechanisms that may regulate Aβ levels in the brain. According to the peripheral sink hypothesis, Aβ Abs in the plasma extract Aβ via equilibrium in efflux of Aβ across the blood-brain barrier (BBB) [14]. In the brain parenchyma, the reduction of Aβ burden may be endogenously carried out by astroglia [15,16] in addition to microglia, which have been demonstrated to participate in both deposition of Aβ [2,17] as well as reduction of Aβ burden by phagocytosis or some other mechanisms [18-20]. Under certain circumstances, microglia-mediated Aβ phagocytosis is enhanced after Aβ deposits are opsonized with active or passive immunotherapy, and this Aβ clearance is also associated with alleviation of cognitive defects or AD-related neuropathological changes [10-14,21]. Immunization can also remarkably alleviate cognitive defects without reduction of Aβ burden [22], possibly because of enhanced peripheral clearance or sequestration of soluble Aβ peptides from the brain to blood [14,23-25]. Monoclonal Abs to Aβ have also been shown to inhibit fibrillization of synthetic Aβ peptide *in vitro *[26], thereby preventing the aggregation of Aβ.

IVIG, purified immunoglobulin obtained from the plasma of healthy humans is indicated for the treatment of primary immunodeficiency disorders associated with defects in humoral immunity. In addition, IVIG is used as an anti-inflammatory therapy for many systemic diseases, including diseases affecting the CNS [27]. Recently, a retrospective study suggested that previous IVIG treatment is associated with a reduced risk of developing AD and related disorders [28]. Furthermore, administration of IVIG to eight patients with mild AD led to transient, reproducible, and dose-dependent increases in serum anti-Aβ Ab titers in parallel with increases in plasma Aβ1-40 and Aβ1-42 levels and improvement in memory functions [29].

Naturally occurring Aβ Abs or specific immune complexes containing auto-Abs to Aβ are significantly less frequent in AD patients than those in age-matched controls, suggesting that reduced levels of auto-Abs to Aβ could lead to increased Aβ deposition in AD [30-33]. Recently it was reported that Aβ Abs abundant in human plasma are reactive against oligomeric Aβ, but the reactivity against oligomeric Aβ assemblies declines with age and advancing AD [34]. Furthermore, IgG was detected in the brain and found to bind to brain Aβ deposits while AD patients with high IgG plaque labeling index had reduced plaque burden which was accompanied with an elevated level of phagocytic microglia [35]. IVIG may serve as a potential therapy to compensate the deprivation of naturally occurring Aβ Abs in AD that would alleviate the Aβ-induced toxicity. Alternatively, IgG antibodies unrelated to specific Aβ Abs may have beneficial effects against Aβ toxicity as IgG has been shown to protect brain also against acute brain injuries [36,37]. However, the detailed mechanisms how IVIG treatment improves the AD pathology and cognitive defects are unclear.

The potential and mechanisms-of-action of IVIG as an anti-inflammatory agent for a broad range of diseases is under intensive investigation. IVIG contains naturally occurring auto-Abs, including Abs against Aβ [38,39] which are able to block synthetic Aβ fibrillization and prevent Aβ-mediated neurotoxicity *in vitro *[39]. IVIG has also been shown to dissolve pre-formed synthetic Aβ fibrils *in vitro*, as well as to promote synthetic Aβ uptake in BV-2 microglia, a murine microglia cell line [40]. However, it is still unsolved whether IVIG could actually dissolve natively pre-formed human Aβ deposits in brain and/or enhance the recognition and reduction of these Aβ deposits by primary microglia. Therefore, it is unclear whether the beneficial effects of IVIG observed in earlier *in vitro *studies using synthetic Aβ have true relevance in Aβ deposition and its possible reduction in AD.

In this study we demonstrate that IVIG prevents Aβ toxicity to hippocampal neurons and that the beneficial effect of IVIG may be mediated by direct neuroprotection as well as by enhanced microglia-mediated, but not astrocyte-mediated, clearance of natively formed diffuse human Aβ deposits in the brain. This microglia-mediated clearance of Aβ occurs by a mechanism involving Aβ-Abs present in IVIG and phagocytic degradation of Aβ. In line with the idea that IVIG treatment can result in microglia-mediated Aβ clearance, we demonstrate that peripherally administered IVIG penetrates into the brain parenchyma of transgenic AD mice and selectively binds to Aβ deposits which are co-localized and surrounded with microglia.

## Methods

### Animals

The amyloid precursor protein and presenilin 1 transgenic (APP/PS1) breeder mice were obtained from Johns Hopkins University, Baltimore, MD, USA (D. Borchelt and J. Jankowsky, Department of Pathology), and a colony was established at the National Laboratory Animal Center, University of Eastern Finland. Briefly, mice were created by co-injection of chimeric mouse/human APP695 harboring the Swedish mutation K595N/M596L and human PS1-dE9 (deletion of exon 9) vectors controlled by independent mouse prion protein promoter elements [41]. The double transgenic mice, APP/PS1, were backcrossed to C57BL6/J for 10-12 generations. Age-matched wild-type littermates served as controls. Animals were housed in a controlled environment, and food and water were available *ad libitum*. Animal experiments were conducted according to the Council of Europe legislation and regulations for animal protection and approved by the Animal Experiment Committee in State Provincial Office of Southern Finland.

### IVIG

Purified IVIG (trade name Gammagard Liquid), prepared from the plasma of healthy humans, was kindly provided by Baxter Innovations GmbH (Vienna, Austria). The control IVIG, depleted of anti-Aβ Abs with affinity chromatography, was also provided by Baxter. The concentration of Aβ Abs is approximately 0.2% of all IgGs present in IVIG [42]. The anti-Aβ depleted IVIG was determined to contain about 5% of its naturally occurring Aβ Abs. The molarity of IVIG was counted based on the molecular weight of Ig, 150 000 Da.

### Aβ neurotoxicity in hippocampal neuronal culture

Primary hippocampal neuronal cultures from E18 C57BL mouse brains were prepared as described previously [43,44]. Briefly, after dissection and papain-dissociation the hippocampi were suspended in Dulbecco's modified eagle medium (DMEM), 10% FBS with penicillin-streptomycin (Gibco, Invitrogen) and plated on poly-DL-ornithine-precoated (0.5 μg/μl; Sigma) 48-well culture plates at 150 000 cells/cm^2 ^and cultivated in humidified atmosphere at 37°C in 5% CO_2_. The next day the medium was changed to serum-free Neurobasal culture medium supplemented with 2% B27, 500 μM glutamine, 25 μM glutamate, and penicillin-streptomycin (Gibco, Invitrogen). To obtain ~90% pure neuronal culture, cells were treated with 10 μM cytosine arabinoside (AraC, Sigma) at days 2-4 to prevent proliferation of other cell types. Thereafter, the whole medium was changed for supplemented Neurobasal to remove AraC, and in addition, one-third of the medium was changed every 3-4 days for maintenance. The hippocampal neurons were used for experiments after 11 days in vitro (DIV).

Aβ1-42 (American Peptide) was dissolved to a stock solution of 1 mg/ml in sterile water. Hippocampal neurons were co-treated with freshly dissolved Aβ1-42 and IVIG for 24 h. Thereafter, the medium was collected and analyzed for lactate dehydrogenase (LDH) release (Sigma) according to the kit protocol. LDH assay measures membrane integrity as a function of the amount of cytoplasmic LDH released into the medium. Hippocampal neurons were fixed with 4% formaldehyde and stained with the bisbenzimide Hoechst 33342, (Sigma) for detection of apoptotic/necrotic cells. The cell viability was determined under the fluorescent microscope (Olympus IX71 microscope with MT10 illumination system attached to DP70 digital camera, running DP software, Olympus) based on the absence of condensed chromatin.

### Aβ fibrillization

Aβ1-42 was dissolved to a stock solution of 1 mg/ml in sterile water. We have previously shown that Aβ1-42 starts immediately and spontaneously to oligomerize and eventually fibrillize [44], being most toxic in oligomer-rich form immediately after being dissolved. To obtain fully fibrillized Aβ, the dissolved peptide was incubated at 37°C for 24 h. The oligomerization state of Aβ was analyzed with immunoblotting for human Aβ (clone 6E10, Signet, Covance) after cross-linking the samples with glutaraldehyde as described previously [44]. The fibrillary state of Aβ has been confirmed with electron microscopy as described previously [44].

The effect of IVIG on Aβ fibrillization was studied by incubating freshly solubilized 10 μM Aβ1-42 in the presence or absence of 5, 10 and 30 μM IVIG or 30 μM of irrelevant human recombinant IgG (Baxter) as a control, at 37°C for 24 h. PBS buffer was used as an additional control. The concentration of the fibrillar Aβ was quantified fluorometrically using Thioflavin-T staining [45]. The samples were added to 2 μM Thioflavin-T (Sigma) solution in 50 mM glycine, pH 9.2. Fluorescence was measured at excitation and emission wavelengths of 435 and 485 nm respectively.

To study whether IVIG could solubilize natively pre-formed Aβ deposits in brain, cryostat-cut brain sections of aged (19 month old) APP/PS1 mice were incubated in the presence or absence of 20 μM IVIG in *ex vivo *medium consisting of X Vivo 15 (Lonza), penicillin-streptomycin and 2 mM L-glutamine (Gibco, Invitrogen) for 7 days after which the medium was collected and the Aβ1-42 concentration was determined with Aβ1-42 ELISA (Biosource). The brain sections were fixed with 4% formaldehyde in PBS for 30 min and analyzed for Aβ content. Nonspecific binding sites were blocked with 10% normal goat serum (NGS) in 0.1% PBS-T. The sections were reacted with pan-Aβ Ab 3 μg/ml (Biosource) in 1% NGS PBS-T overnight, followed by 10 μg/ml Alexa568 secondary Ab (Molecular Probes) for 2 h at room temperature (RT). The glass coverslips were mounted onto microscope slides using Vectashield containing nuclear stain DAPI (Vector Laboratories). The sections were imaged with an Olympus AX70 microscope attached to a digital camera (Color View 12 or F-View, Soft Imaging System) running an Analysis Software (Soft Imaging System) and quantified (Image ProPlus, Media Cybernetics) for the Aβ burden as an indicator of solubilization of pre-formed Aβ deposits.

### Degradation of brain Aβ by primary microglia cells

Mixed microglia cell culture was prepared from P0-P1 C57BL mouse pups as described [46]. Briefly, the cortices and midbrain were dissected out and meninges were removed. The tissues were dissociated with trypsin and the cells were eventually resuspended into DMEM, 10% FBS, 100 U/ml penicillin-streptomycin and 2 mM L-glutamine (Gibco, Invitrogen). The cells were plated on cell culture flasks coated by poly-L-lysine (PLL, Sigma) and cultivated in humidified atmosphere at 37°C in 5% CO_2_. The medium was changed after two days and thereafter every 2-3 days of cultivation. The loosely attached microglia were harvested after 12 DIV by shaking the flasks at 120 rpm for 10-15 min at 37°C in an orbital shaker. For *ex vivo *experiments, microglia were resuspended in serum-free X Vivo 15 medium (Lonza) supplemented with penicillin-streptomycin and 2 mM L-glutamine.

The *ex vivo *Aβ degradation assay was modified from Koistinaho et al. [15]. Aged APP/PS1 mice were perfused with heparinized saline and brain hemispheres were frozen on dry ice. Cryostat-cut (Leica) 10-μm-thick sagittal brain sections were mounted on PLL-coated glass coverslips and transferred onto 24- or 48-well cell culture plates and stored at -20°C until use. Brain sections were thawed shortly before use and incubated with IVIG for 1 h at 37°C, after which the cells were applied onto the brain sections, 250 000 cells/cm^2 ^with or without IVIG. After 24 h of incubation, the sections were fixed and immunostained as described above. The sections were quantified as described above for the amount of remaining Aβ immunostaining (Aβ burden) as an indicator for Aβ clearance by the cells. IVIG concentrations higher than 20 μM may cause an excess dilution of the cell culture medium and may not be relevant for *in vivo *conditions. Thus, IVIG concentrations ≤20 μM were used.

To study IVIG interaction with Aβ deposits in microglia-mediated Aβ clearance, the brain sections were first incubated with IVIG for 1.5 h at 37°C and after wash out of any unbound IVIG, the microglia were applied as described above. After 24 h of incubation, reduction of Aβ burden was quantified as described above.

To elucidate the contribution of and the effect of IVIG on lysosomal degradation, the major intracellular protein degradation pathway in microglia-mediated Aβ clearance, microglia *ex vivo *assay with or without IVIG was performed in the presence of 500 nM bafilomycin A1 (Baf) (Sigma) to inhibit the lysosomal degradation pathway in microglia. After 24 h of incubation, the reduction of Aβ burden was quantified.

The analysis of co-localization of Aβ deposits and microglia was performed by imaging the same site of the brain section after exciting Alexa568 and DAPI fluorescence, respectively. The figures were merged with Adobe Photoshop. The count the number of microglia the same site of sections were imaged as described above and the cells were counted based on the nuclear stain DAPI present in the mounting medium.

Cell viability was determined by resazurin assay. After 24 h of incubation, 10 μM resazurin (Sigma) was applied into cell culture medium and incubated for 4 h. Medium samples were collected into 96-well plate and measured with excitation 544 nm, emission 590 nm (Victor Wallac).

### Degradation of brain Aβ by primary astrocytes

Adult astrocyte cell culture was prepared as described before [15]. Briefly, hippocampi and cortices were isolated from 6-8-week-old C57BL mice and the tissues were dissociated with trypsin followed by Percoll (Sigma) centrifugation. The cells were eventually resuspended into DMEM/F12, 10% FBS, 100 U/mL penicillin-streptomycin and G5 supplement (Gibco, Invitrogen), plated onto PLL-coated cell culture flasks and cultivated in humidified atmosphere at 37°C in 5% CO_2 _for several passages. Before the experiments the glial cell cultures were shaken at 200 rpm for 2 h at 37°C to remove microglia.

For *ex vivo *experiments, astrocytes were re-suspended in serum-free DMEM/F12 medium, 0.2% BSA (Sigma), 100 U/ml penicillin-streptomycin and G5 supplement and applied onto the brain sections at 150 000 cells/cm^2 ^as described above.

### Penetration of IVIG into the brain

To study the brain access of human IVIG, 4-month-old APP/PS1 mice or their wild-type littermate controls received i.p. injections of 1.0 g/kg of 10% IVIG or equal volume (10 ml/kg) of saline. The short-term injections (1-3 weeks) were administered twice a week while the long-term injections were given once per week.

### Intrahippocampal injection of IVIG

Intrahippocampal injections were performed as previously described [47]. Briefly, IVIG was injected unilaterally (200 μg in the volume of 2 μl) into the hippocampi of 16-month-old APP/PS1 mice. The animals were transcardially perfused 3 days later, and the brains were applied to immunohistochemistry.

### Immunohistochemistry

At the end of the study the mice were anesthetized with an anesthetics cocktail consisting of 105 mg/kg pentobarbiturate and 425 mg/kg chloral hydrate, and transcardially perfused with heparinized saline. Brains were retrieved rapidly and immersion fixed in 4% paraformaldehyde in 0.1 M PB for 4 h, moved to 30% sucrose in 0.1 M PB overnight and stored at -70°C until further processed. Coronal brain sections, 35 μm in thickness, were cut with a freezing-sliding microtome at the level of the septal (dorsal) hippocampus. For human IgG staining the sections were treated with 0.1% H_2_O_2 _in methanol, and incubated overnight with rabbit anti-human HRP-labelled Ab (DAKO, 1:7500 in TBS containing 0.25% Triton X-100 (TBS-T), pH 7.6. Immunoreactivity was visualized with H_2_O_2 _and 0.25% nickel enhanced diaminobenzidine (Sigma).

For triple staining to study co-localization of Aβ, microglia and human IgG, free floating sections were extensively washed with 0.1 M TBS, pH 7.4 and non-specific binding sites in the tissue were blocked with 5% normal donkey serum in TBS, to which 0.3% Triton X-100 had been added (NDS-TBS-T), for 1 h. The sections were then incubated overnight with a mixture of biotinylated monoclonal mouse anti-Aβ17-24 (clone 4G8, Covance, 1:100 in NDS-TBS-T) and rabbit-anti-Iba 1 (Wako Chemicals GmbH, Neuss, Germany, 1:400). Next, the sections were rinsed with TBS and applied to a cocktail of highly purified, carbocyanine (Cy)-conjugated secondary Abs (all from Jackson ImmunoResearch, West Grove, PA, 20 μg/ml in TBS containing 2% BSA): Cy3-coupled streptavidin, Cy5-coupled donkey-anti-rabbit IgG and Cy2-coupled donkey anti-human IgG. The sections were examined with a confocal laser-scanning microscope (LSM10 Meta, Zeiss) as described before [48]. The Cy5-immunolabelling was color-coded in blue.

### Statistical analysis

The data are expressed as mean ± SD and were analyzed with SPSS software using Student's T-test or one-way ANOVA when appropriate, followed by Dunnett's or Tukey's *post hoc *test. * (p < 0.05), ** (p < 0.01), *** (p < 0.001).

## Results

### IVIG protects primary hippocampal neurons against Aβ1-42 toxicity

**Figure 1:**
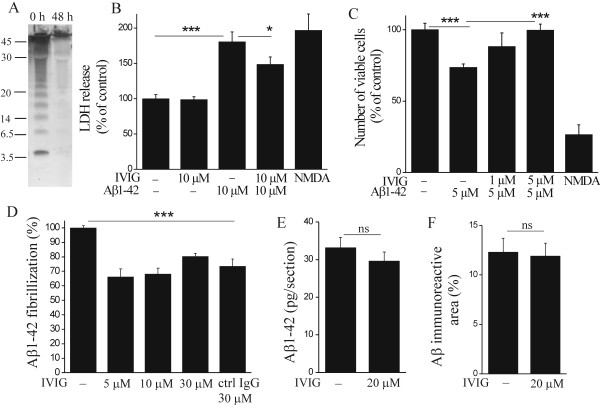
**IVIG protects primary hippocampal neurons against Aβ1-42 toxicity**. Freshly solubilized 5 μM Aβ1-42 (Aβ oligomer rich preparation) was incubated for 0 or 48 h, cross-linked to preserve the peptide structure and analyzed with western blotting with an anti-Aβ antibody (6E10). Freshly solubilized Aβ1-42 consisted of monomers, dimers, various forms of oligomers and aggregates with larger molecular weight (Mw). After 48 h of incubation, low Mw forms of Aβ1-42 were reduced while high Mw aggregates were detected (A). Mw of Aβ1-42 monomer is 4.5 kDa. Mouse hippocampal neurons were exposed to freshly solubilized Aβ1-42. After 48 h of incubation, the cytotoxicity was determined with a defect in cell membrane integrity and the presence of condensed chromatin in nuclei. Aβ1-42 was highly cytotoxic (B, C) in micromolar concentrations (n = 3, p < 0.001). IVIG reduced Aβ1-42-induced neurotoxicity by the terms of cytoplasmic LDH release (B, n = 3, p < 0.05) and the number of apoptotic/necrotic cells (C, n = 3, p < 0.001). The effect of IVIG on Aβ fibrillization was studied by incubating freshly solubilized 10 μM Aβ1-42 in the presence of IVIG or irrelevant human recombinant IgG (ctrl IgG) for 24 h and quantifying fibrillar Aβ fluorometrically with Thioflavin-T staining. IVIG reduced Aβ1-42 fibrillization (D, p < 0.001, n = 3) but this was not concentration-dependent and also occurred in the presence of an irrelevant IgG (D, p < 0.001, n = 3). To study the effect of IVIG on solubilization of natively formed brain Aβ deposits, unfixed cryostat-cut hippocampal brain sections prepared from aged APP/PS1 mice were incubated with 20 μM IVIG for 7 days. IVIG did not solubilize pre-formed Aβ deposits; the release of Aβ into the medium was not increased (E, p > 0.05, n = 6) while Aβ immunoreactive area in the brain sections was not decreased (F, p > 0.05, n = 6), as determined with ELISA and immunostaining, respectively.

When mouse hippocampal neuronal cultures containing 90% of neurons were exposed to 5 or 10 μM oligomer-rich Aβ1-42 preparation (Figure 1A) for 48 h, significant neuronal death was observed by LDH release into the cytoplasm and by counting surviving neurons (p < 0.001, Figure 1B and 1C). Co-administration of 10 μM IVIG reduced the toxicity of 10 μM Aβ1-42, when LDH release was measured (p < 0.05, Figure 1B), and 5 μM IVIG completely blocked the toxicity of 5 μM Aβ1-42, when viable cell count was recorded (p < 0.001, Figure 1C). IVIG treatment alone posed no toxicity to primary neuronal cells (Figure 1B).

### IVIG prevents Aβ1-42 fibrillization to the same extent as irrelevant IgG and does not solubilize natively formed brain Aβ deposits

Potential mechanisms for IVIG-induced neuroprotection against Aβ-induced cell death include interaction of IVIG with Aβ1-42 peptides by preventing oligomerization and further fibrillization of Aβ1-42, or solubilization of pre-formed Aβ1-42 fibrils/aggregates. When Aβ1-42 was taken into aqueous solution, Aβ1-42 started to oligomerize immediately, reaching a fully aggregated state after 48 h of incubation (Figure 1A) as we have also previously shown [44].

When 10 μM Aβ1-42 was incubated in the presence of IVIG, fibrillization of Aβ1-42 was reduced as determined with Thioflavin-T staining (Figure 1D). However, the effect of IVIG on Aβ1-42 fibrillization was not concentration-dependent and did not differ from the effect of irrelevant human IgG. This suggests that the reduction of Aβ1-42 fibrillization was not due to any specific immunoglobulins present in IVIG.

To test whether IVIG could solubilize natively formed human Aβ deposits in brain, sections of aged APP/PS1 mouse brains were incubated with 20 μM IVIG for seven days and the medium was collected for quantification of solubilized Aβ1-42 with ELISA. In addition, the brain sections were analyzed for the possible reduction of Aβ burden in the tissue by Aβ immunostaining. We observed no effect of IVIG on the amount of Aβ found in the media as an indicator of the solubilization of Aβ1-42 from the brain sections (Figure 1E) or on total Aβ burden in the brain sections (Figure 1F).

### IVIG specifically promotes microglia-mediated clearance of brain Aβ

**Figure 2:**
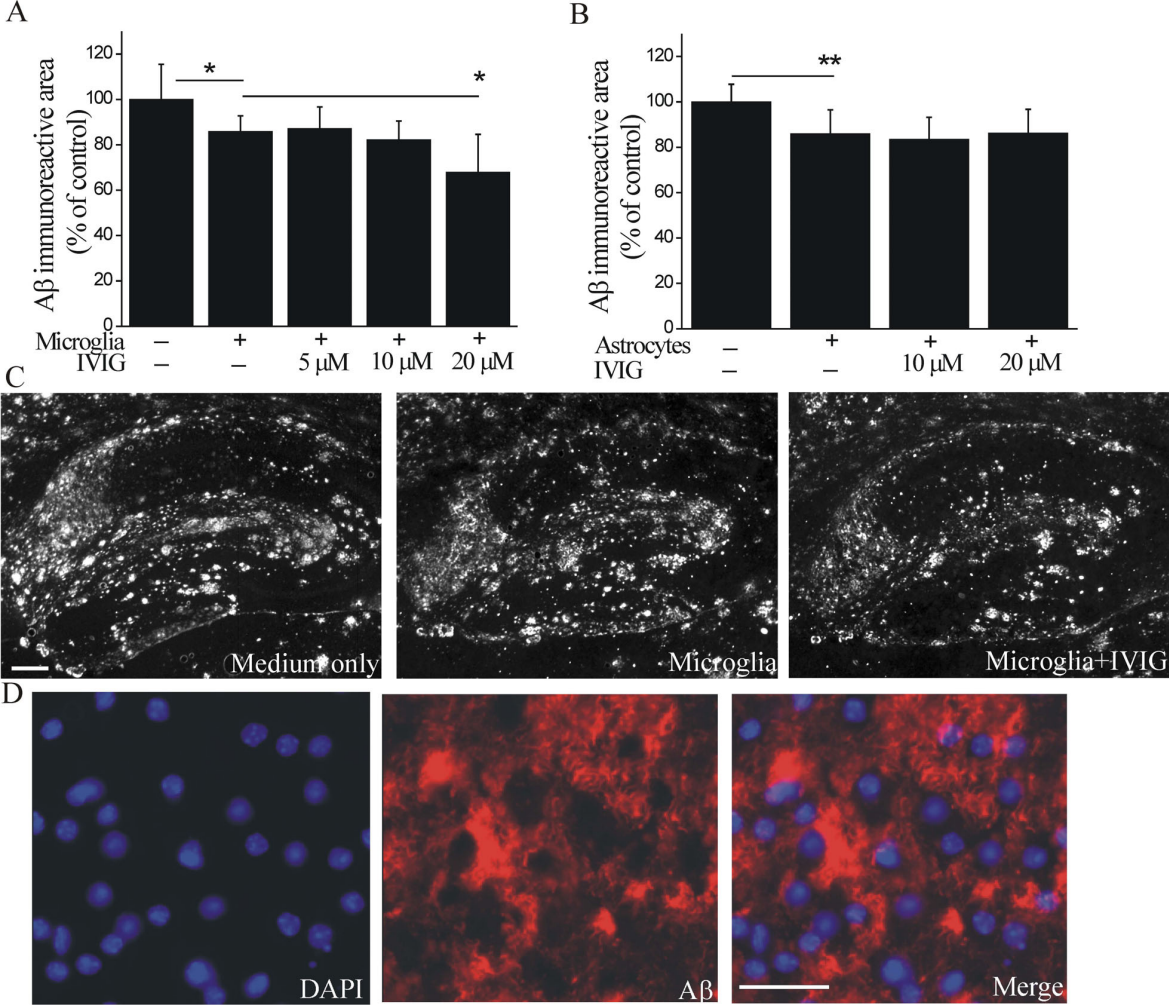
**IVIG promotes microglia-mediated clearance of natively formed Aβ deposits**. In an *ex vivo *assay, neonatal mouse microglial cells were incubated on top of APP/PS1 brain sections. After incubation period of 24 h, the Aβ burden was quantified by Aβ immunostaining. Microglia alone reduced Aβ burden *ex vivo *(A, C, n = 6-8, p < 0.05) as quantified from the whole hippocampus area. IVIG further promoted microglia-mediated Aβ clearance (A, C, n = 6-8, p < 0.05). The reduction of Aβ was observed as Aβ-free cavities in the brain sections at sites of cultured microglia cell bodies, visualized with DAPI, as exemplified for the subiculum (D). When the *ex vivo *assay was performed for adult mouse astrocytes, we observed a significant reduction of Aβ burden (B, n = 6-8, p < 0.01), quantified from the whole hippocampus area, but no promotion of Aβ clearance with up to 20 μM IVIG (B, n = 6-8). Scale bars 200 μm (C) and 50 μm (D).

When microglia were applied on top of unfixed cryostat-cut brain sections prepared from aged APP/PS1 mice, and Aβ burden was quantified as Aβ immunoreactivity covering the hippocampal area of the brain section, we observed a reduction of human Aβ burden to 86% when compared to control sections (p < 0.05, Figure 2A and 2C). The analysis was focused on hippocampal brain areas of the brain sections due to region-specific and even distribution of human Aβ within those areas, making comparisons between the sections feasible. Twenty micromolar IVIG further promoted the ability of microglia to reduce Aβ burden to 68% from that of control sections (p < 0.05) (Figure 2A and 2C). Based on the co-localization of Aβ immunoreactivity and DAPI nuclear staining, the reduction of Aβ was also seen as Aβ-free cavities in the brain sections at sites of cultured microglia cell bodies (Figure 1D). IVIG *per se *did not interfere with the subsequent detection of Aβ immunoreactivity (total Aβ burden 11.3 ± 0.8% in control and 10.8 ± 1.1% in 20 μM IVIG treated sections, p > 0.05), confirming that the reduction of Aβ immunoreactivity indeed represents the reduction of Aβ burden in the brain tissue.

Primary astrocytes obtained from adult but not neonatal mouse brain has been shown to participate in the removal of Aβ from the brain [16]. When the *ex vivo *assay was performed with adult mouse astrocytes, a significant reduction of Aβ burden to 86% of control (Figure 2B p < 0.01) was observed, as previously described [15]. However, IVIG up to 20 μM concentration did not promote the astrocyte-mediated Aβ clearance (Figure 2B). The inability of IVIG to enhance astrocyte-mediated Aβ clearance was not due to prevention of astrocyte clustering which has been previously shown [15] to be required for Aβ clearance by these cells (data not shown).

**Figure 3:**
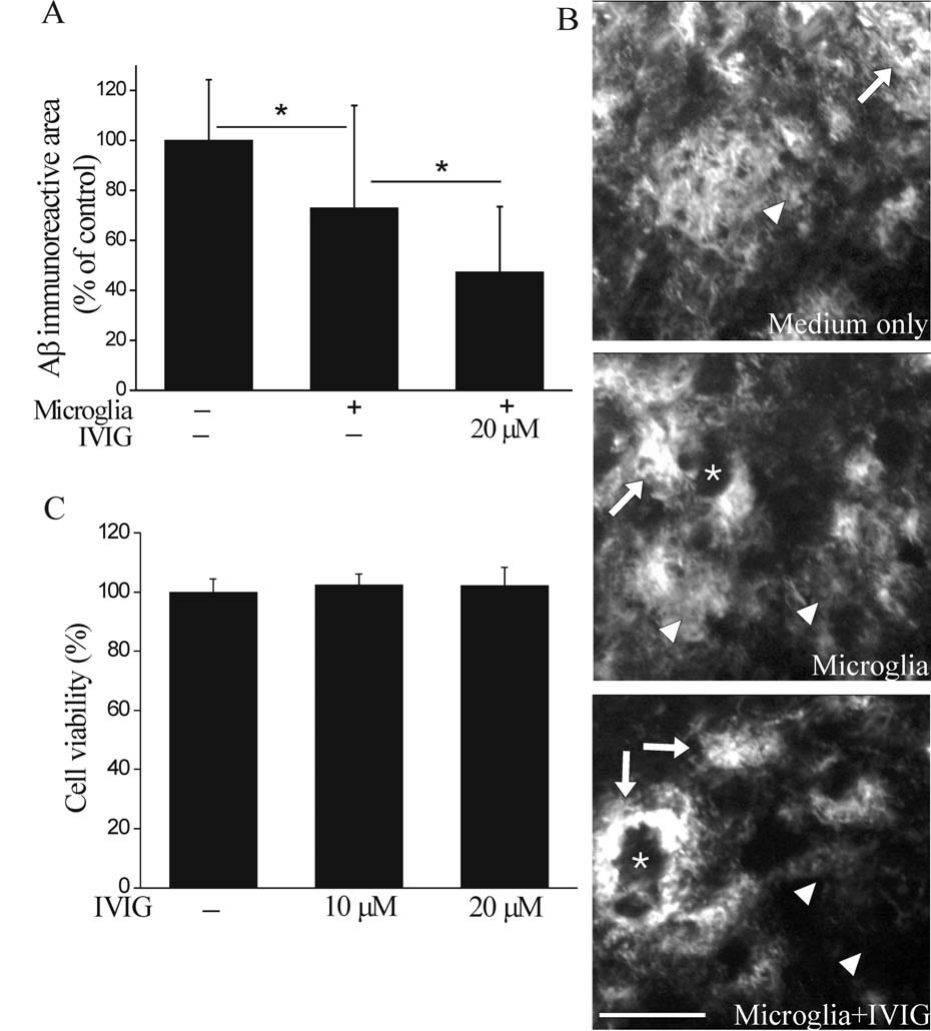
**IVIG promotes miroglia-mediated clearance of diffuse Aβ deposits**. Within the hippocampus the microglia-mediated reduction in Aβ burden was the most evident in the subiculum which displayed both dense (arrows) and diffuse (arrowheads) Aβ deposition (B). We observed primary microglia to reduce Aβ burden from the subiculum (A, n = 15, p < 0.05). IVIG further promoted the microglia-mediated clearance of Aβ (A, n = 15, p < 0.05). Diffuse Aβ deposits (arrowheads) were greatly reduced and nearly disappeared when incubated in the presence of microglia and 20 μM IVIG (B). Aβ-free cavities, present in the brain sections incubated with microglia or microglia and IVIG, are shown with asterisks (*). IVIG did not affect cell proliferation or viability as measured with cellular metabolic activity (C). Scale bar 50 μm.

The microglia-mediated reduction in brain Aβ burden was more evident in regions containing the highest amount of Aβ-immunoreactive material, particularly in the subiculum, which showed accumulation of both diffuse and more dense Aβ deposits. Quantifying immunoreactivity in the subiculum, we observed that microglia reduced Aβ burden to 73% of control sections (p < 0.05, Figure 3A). Twenty micromolar IVIG further promoted the microglia-mediated reduction of Aβ burden to 47% (p < 0.05, Figure 3A). The reduction of Aβ was also observed as Aβ-free cavities in the sections at sites of microglia cell bodies (Figure 3B). Furthermore, we discovered that diffuse Aβ immunoreactivity was greatly reduced and nearly disappeared when incubated in the presence of microglia and 20 μM IVIG (Figure 3B).

To exclude the possibility that IVIG induced microglia proliferation and thereby enhanced microglia-mediated clearance of Aβ, we counted the microglia cells at the 24 h time point (data not shown) at the end of the *ex vivo *assay from images taken exactly from the same site of the brain section that was used to quantify Aβ clearance. The enhanced clearance of Aβ was not due to the increased number of microglia in response to IVIG. Moreover, IVIG had no effect on cell viability as studied with resazurin assay measuring cellular metabolism (Figure 3C).

### Mechanisms of microglia-mediated clearance of brain Aβ

**Figure 4:**
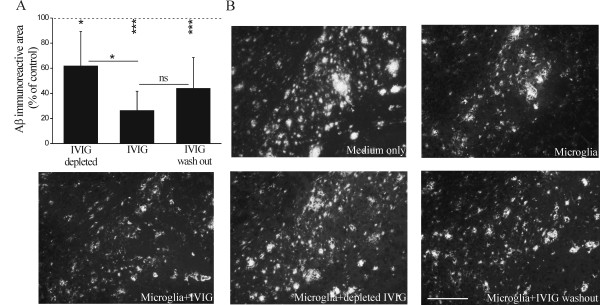
**Promotion of microglia-mediated clearance of Aβ is dependent on Aβ antibodies in IVIG**. The depletion of Aβ antibodies from IVIG significantly compromised the IVIG-promoted microglia-mediated Aβ clearance (A, n = 5-6, p < 0.05) as imaged in the subiculum (B). Preincubation of brain sections with 20 μM IVIG followed by wash out of any unbound IVIG prior to addition of exogenous primary microglia conserved microglia-mediated Aβ clearance (A, B, n = 5-6, p < 0.001) that did not significantly differ from the *ex vivo *incubation in the presence of IVIG throughout the assay (n = 5-6, p > 0.05). Dotted line represents the Aβ immunoreactive area in intact hippocampal brain sections incubated without microglia. The statistical significance of Aβ clearance in comparison to intact brain section incubated without microglia is shown as asterisks below the dotted line, indicating the respective p-values. Scale bar 100 μm.

We next examined whether the effect of IVIG on Aβ clearance was dependent on anti-Aβ Abs present in IVIG. The *ex vivo *assay was therefore performed in the presence of IVIG depleted for Aβ Abs (depleted IVIG). Microglia were able to reduce Aβ burden in the presence of IVIG and depleted IVIG to 26% (p < 0.001) and 62% (p < 0.05) of control, respectively (Figure 4A and 4B). However, the microglia-mediated clearance of Aβ was significantly diminished (p < 0.05) in the presence of depleted IVIG in comparison to IVIG, suggesting that Aβ Abs present in IVIG are further promoting Aβ clearance (Figure 4A). To reveal whether binding of certain components of IVIG preparation was enough to induce enhanced Aβ clearance by microglia, the pre-incubation of brain sections with IVIG was followed by wash out of any unbound IVIG prior to addition of microglia reduced Aβ burden to 44% (p < 0.001) of control (Figure 4A). In addition, following IVIG wash out microglia did not significantly differ from IVIG-treated microglia in their capacity to reduce Aβ burden. These results suggest that interaction of IVIG with Aβ deposits may be sufficient to promote Aβ clearance by microglia (Figure 4A and 4B).

**Figure 5:**
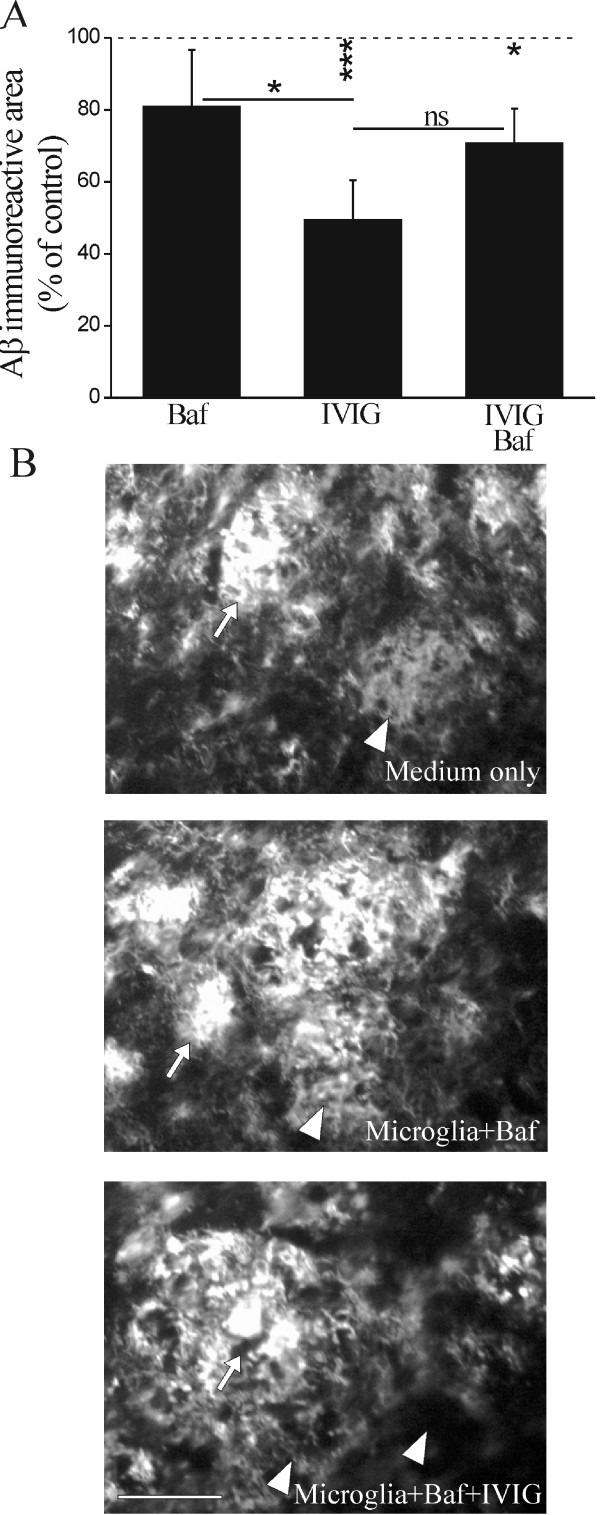
**Promotion of microglia-mediated clearance of Aβ is dependent on lysosomal degradation**. To study the role of lysosomal degradation in microglia-mediated Aβ clearance, the *ex vivo *assay was performed in the presence of Baf, an inhibitor of lysosomal degradation pathway. Baf inhibited microglia-mediated Aβ clearance (A, n = 3-6) as Aβ deposits remained mostly intact in sections treated with Baf (B). IVIG could partially restore microglia-mediated Aβ clearance halted by Baf. In the presence of Baf, 20 μM IVIG treatment resulted in low clearance and mostly of diffuse Aβ deposits only (A, B, n = 3-4, p < 0.05). Dense (arrows) and diffuse (arrowheads) Aβ deposits are shown in the subiculum. Scale bar 50 μm.

To study the role of lysosomal degradation in microglia-mediated Aβ clearance, we performed the *ex vivo *assay in the presence of Baf, an inhibitor of vacuolar H^+ ^ATPase (V-ATPase). Baf disrupts the lysosomal membrane proton pump that maintains the low intralysosomal pH needed for normal lysosomal activity [49]. In the presence of both Baf and microglia, Aβ deposits remained mostly intact showing that intralysosomal pH and anticipated inactivation of lysosomal proteases by Baf prevented the ability of microglia to clear brain Aβ (p > 0.05, Figure 5A and 5B). IVIG was partially able to restore microglia-mediated Aβ clearance halted by Baf as IVIG-enhanced microglia-mediated clearance of Aβ did not significantly decay when Baf was administered (p > 0.05, Figure 5A). In the presence of Baf, IVIG enhanced microglia-mediated Aβ clearance predominantly from the sites of diffuse Aβ deposits (Figure 5B). These results suggest that the IVIG-enhanced Aβ clearance involves Aβ uptake by microglia and is dependent on intracellular lysosomal degradation.

### IVIG penetrates into the brain and specifically binds Aβ deposits

**Figure 6:**
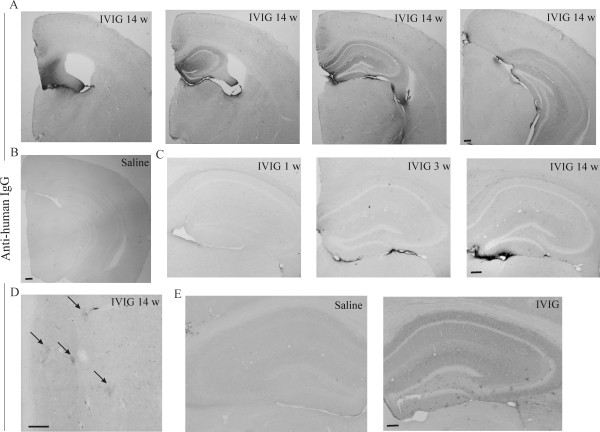
**Human IgG immunoreactivity is detected in the brain after peripheral administration of IVIG**. To study whether IVIG could penetrate the BBB to reach brain parenchyma, APP/PS1 mice were injected i.p. with IVIG 1 g/kg or saline starting at the age of 4 months. The IVIG was identified from cryostat-cut mouse brain sections with anti-human IgG. IVIG penetrated BBB as evidenced with intensive human IgG staining in the mouse hippocampus (A) with a clear anterior-posterior gradient with the highest immunoreactivity at the septal end of the hippocampus adjacent to the choroid plexus. Also, strong immunoreactivity was observed lining the ventricles (A). The intensity of human IgG immunoreactivity was increased with the duration of IVIG treatment from 1 to 14 weeks of IVIG admininistration (C). Saline-treated mice were devoid of specific immunoreactivity for anti-human IgG (B). In the hippocampus, Aβ deposits appeared as stippled patterns of aggregated material covered with immunoreactivity for human IgG (black arrows) (D). After a single intrahippocampal injection of IVIG the IgG immunoreactivity was spread homogeneously throughout the hippocampus (E) while stronger IgG immunoreactivity was observed as stippled patterns (E). Scale bars 200 μm (A, B, E) and 50 μm (D).

Finally, we tested whether the cellular mechanisms of Aβ clearance observed *in vitro *have potential relevance *in vivo *and dosed a few APP/PS1 mice i.p with IVIG or saline starting at the age of 4 months. The concern was whether human IVIG is able to cross the BBB to reach brain parenchyma in effective concentrations. After transcardial perfusion with saline to abolish any possible interference with IVIG present in the blood, the IVIG was identified from mouse brain sections with anti-human IgG. IVIG penetrated the BBB as evidenced with intensive anti-human IgG staining in the mouse hippocampus (Figure 6A). There was a clear anterior-posterior gradient with the highest immunoreactivity at the septal end of the hippocampus adjacent to the choroid plexus. In addition, strong immunoreactivity was observed lining the ventricles (Figure 6A). The intensity of anti-human IgG immunoreactivity increased with the duration of IVIG treatment (Figure 6C). Human IgG immunolabeling was also observed in wild-type mice, but to a lesser extent, suggesting that the BBB is more leaky in APP/PS1 mice (data not shown). When few APP/PS1 mice were injected with IVIG i.v., stronger immunoreactivity was detected in the hippocampus than after i.p. administration, suggesting that a higher concentration of IVIG available in the blood leads into a higher rate of penetration through the BBB, favoring the i.v. administration route (data not shown).

**Figure 7:**
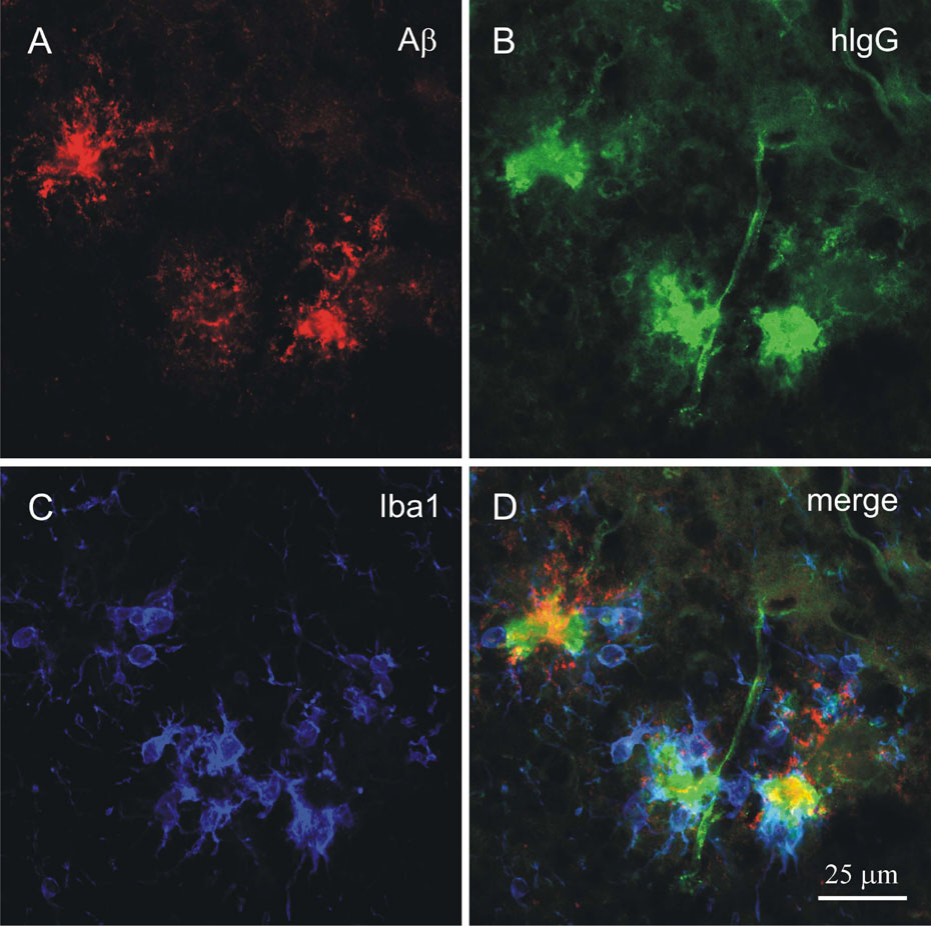
**IVIG interacts with human-type Aβ brain deposits in APP/PS1 mouse brain after peripheral administration**. Triple immunofluorescence labelling of Aβ deposits, human IgG and microglia was performed after peripheral i.p. administration of IVIG. Aβ-immunoreactive deposits (A) and human IgG (B) was observed to co-localize in the brain parenchyma, as revealed by confocal laser-scanning microscopy imaged in the lateral entorhinal cortex (D). Human IgG was detected predominantly within Aβ plaques, but also in blood vessels (B). Human IgG-bound Aβ deposits were surrounded by Iba-1 positive microglia (C, D). Merging the immunosignals in (D) indicates that Aβ deposits were targeted both by human IgG and recruited microglia. The omission of primary antibodies resulted in the expected absence of immunolabelling.

In the hippocampus, Aβ deposits appeared as stippled patterns of aggregated material covered with human IgG immunoreactivity (Figure 6D), which was not observed in IVIG treated wild type mice. When IVIG was injected directly into the hippocampus, homogenous IgG immunoreactivity was observed (Figure 6E) similar to the IgG immunoreactivity observed after the peripheral IVIG administration (Figure 6A). After the local intrahippocampal injection, IgG immunoreactivity was defined within the injected brain hemisphere and IgG immunoreactivity was not observed lining the ventricles (Figure 6E). After intrahippocampal injection, IVIG appeared as stippled patterns of aggregated material covered with human IgG immunoreactivity (Figure 6E). Some human IgG immunoreactive spots were also positive for Congo Red staining, as a label for β-sheet structures in dense core Aβ deposits (data not shown). Finally, we verified whether IVIG interacts with brain deposits of human-type Aβ in transgenic mice after peripheral administration of IVIG. A closer examination with confocal microscopy revealed co-localization of human IgG and Aβ deposits (Figure 7A, B and 7D), surrounded by increased number of microglia (Figure 7C and 7D). Very low levels of IVIG in areas devoid of Aβ suggests a highly specific interaction of IVIG with Aβ deposits *in vivo*.

## Discussion

IVIG has been proposed as a potential therapy for AD and it has recently been shown to alleviate AD pathology in an 18-month study carried out with 8 patients with mild AD [29]. However, the exact mechanism how IVIG treatment may improve the AD pathology is unclear. Our study demonstrates that - in addition to neuroprotection towards Aβ toxicity - IVIG enhances microglia-mediated but not astrocyte-mediated Aβ clearance. Immunoglobulins in general seem to reduce Aβ toxicity, and this beneficial effect is significantly enhanced by Aβ specific Abs. We demonstrate that peripherally administered IVIG penetrates into the brain of transgenic mice mimicking human AD pathology and selectively binds to Aβ deposits in the brain parenchyma. Aβ deposits co-localized with IVIG are also tightly surrounded with Iba-1 positive microglia, demonstrating notable relevance *in vivo*.

The accumulation of Aβ within the brain of AD patients reflects an imbalance between the deposition of Aβ and its clearance from the brain parenchyma, leading to pathological events including neurotoxicity and inflammation. We performed neurotoxicity studies with freshly solubilized Aβ1-42, modeling Aβ toxicity in the presence of Aβ oligomers and fibrils, as we and others have described before [44,50]. IVIG or purified Aβ Abs of IVIG have been shown to inhibit neurotoxicity induced by Aβ oligomers in rat cortical neurons [39] and N2A secondary cells [51]. We demonstrate IVIG to reduce Aβ1-42 induced neurotoxicity in primary mouse neuronal cells cultured from the hippocampus, a brain area typically affected in AD. Britschgi et al. [34] reported that IgG purified from an individual AD patient or a healthy non-demented control reduced Aβ neurotoxicity in a similar manner.

Aβ deposits are reduced in transgenic mouse models of AD *via *several mechanisms: (i) the inhibition of Aβ fibrillization by Ab binding to Aβ [26,52], (ii) glial cell mediated clearance of Aβ, including microglia-mediated phagocytosis of opsonized Aβ deposits [13,21,53], and (iii) the peripheral sink route in which Aβ Abs in the plasma extract Aβ via equilibrium into the efflux of Aβ across the BBB [14]. It should be noted here, that different origin of IVIG preparations may account for binding of Aβ species to varying extent [54,55] causing a possible source of variation between different studies. Abs purified from IVIG have been shown *in vitro *to inhibit the fibrillization as well as to dissolve the preformed fibrils of Aβ25-35, Aβ1-40 and Aβ1-42 [39]. In addition, IVIG itself without further purification steps was shown to dissolve pre-formed Aβ1-40 fibrils [39]. We have widely used an Aβ1-42 oligomerization and fibrillization model in our previous Aβ neurotoxicity studies [44,56,57]. Utilizing this model, we observed IVIG to modestly reduce synthetic Aβ1-42 fibrillization as detected with Thioflavin-T stain. This finding is in line with previous studies with IVIG in Aβ1-42 fibrillization [39]. However, we found this was not due to any specific components present in IVIG since it also occurred upon application of an irrelevant human recombinant control Ab, suggesting a mechanism independent of Aβ Abs and possibly partly characteristic to Ab fragments in general. Furthermore, we observed no effect of IVIG on solubilization of natively formed human Aβ deposits from APP/PS1 mouse brain sections. Besides detecting no solubilization of Aβ into the medium, we observed no effect of IVIG on Aβ burden quantified from APP/PS1 mouse brain sections either. Since IVIG had no additional effect on Aβ1-42 fibrillization when compared to irrelevant IgG, we suggest IVIG to have a direct effect on neuronal cells to reduce Aβ1-42 toxicity, or indirect protection *via *neutralization of Aβ1-42.

IVIG has previously been shown to enhance the uptake of exogenously provided fibrillar Aβ in BV-2 cells, a secondary cell line model for microglia [40]. Instead of determining Aβ uptake, we investigated the phagocytosis of Aβ by primary mouse microglia in an *ex vivo *assay, where the clearance of natively deposited brain Aβ was studied. The clearance of Aβ by primary microglia has been shown to occur predominantly after opsonization of Aβ deposits requiring their decoration with Abs for consequent recognition by microglia [10-14,21]. We found primary microglia to reduce Aβ burden without any preceding opsonization step required. Moreover, we discovered IVIG to further enhance the clearance of Aβ deposits in a dose-dependent manner. In addition, the pre-treatment of brain sections with IVIG and wash-out of unbound IVIG before application of microglia was sufficient to enhance the clearance of Aβ deposits. This suggests that interaction of IVIG with the Aβ deposits may be sufficient to promote Aβ clearance by microglia. Furthermore, the microglia-mediated clearance of Aβ was significantly reduced in the presence of depleted IVIG in comparison to IVIG, suggesting that Aβ Abs present in IVIG participated in Aβ clearance. The ability of IVIG to induce promotion of Aβ clearance appears to be specific for primary microglia, since we found no further enhancement of Aβ clearance by primary astrocytes.

Reduction of diffuse Aβ deposits occurred at sites of microglial cell body, where cavities in layers of human Aβ were seen to form. This phenomenon resembled "moth-eaten" Aβ plaques previously described *in vivo *[58] showing special characteristics of microglia-mediated Aβ clearance. Earlier reports suggest that microglia express proteases such as MMPs which could degrade Aβ extracellularly [59-61] and that IVIG induces expression of MMP-9 in microglia [62]. When *ex vivo *brain sections were incubated in the presence of microglia-conditioned and microglia plus IVIG-conditioned medium, we observed no significant reduction of brain Aβ burden (data not shown), suggesting that extracellular proteases secreted by microglia were not the primarily responsible for IVIG-enhanced microglia-mediated Aβ clearance.

Cultured microglia have been shown to internalize Aβ and deliver it to lysosomes [63] where most non-selective protein degradation takes place. Baf, an inhibitor of V-ATPase, disrupts the maintenance of intralysosomal low pH [49]. We observed that Aβ deposits remained mostly intact in sections with Baf, indicating that intralysosomal pH and anticipated inactivation of lysosomal proteases by Baf affected the capability of microglia to clear Aβ. However, even in the presence of Baf, IVIG treatment resulted in microglia-mediated clearance mostly from diffuse Aβ deposits, suggesting that IVIG may potentiate microglia-mediated Aβ clearance by counteracting lysosomal deficits in microglia. In addition, inactivated microglia have weakly acidic lysosomes, and lysosomal acidification during activation of microglia is required for Aβ degradation by microglia [64]. Thus our results suggest that Aβ clearance occurs by lysosomal degradation and boosting the lysosomal activity of microglia, potentially by IVIG, could increase Aβ clearance.

Recently, a receptor for anti-inflammatory activity of IVIG was identified. Sialylated IgG Fc fragments in IVIG were demonstrated to bind to SIGN-R1, a receptor on mouse splenocytes, resulting in secretion of soluble anti-inflammatory mediators that regulate macrophage activity [65]. However, since our model based on isolated microglia from the CNS, this is not likely the mechanism for IVIG activity. Generally, IVIG has been proposed to regulate the activating and inhibitory FcγR on macrophages to modulate inflammation [27]. Since the FcγR-mediated effect would rather suppress the inflammatory pathways, including phagocytosis, this is not likely the mechanism explaining the IVIG-enhanced phagocytosis in microglia. In the presence of IVIG, BV-2 microglia display ramified morphology with high expression of activation marker CD45, suggesting that IVIG induces a specific activation pattern on microglia [40]. Furthermore, it has been shown that intracranial Aβ Ab administration leads into a biphasic clearance of Aβ deposits [66]; first a rapid removal of diffuse Aβ deposits, and second the removal of compact Aβ deposits associated with a transient activation of microglia. We found IVIG to enhance microglia-mediated clearance of diffuse Aβ largely dependent on Aβ Abs.

Unmodified immunoglobulins show a low tendency to penetrate through the BBB [67], but in certain conditions such as specific stages of diseases and in senescence, immunoglobulins have easier access into the brain [13,68]. Natural Abs recognizing oligomeric Aβ assemblies have been found to reside in the human cerebrospinal fluid, but at 30-230 times lower concentration than in plasma but the repertoires of IgG Abs are the same in these two compartment [34]. IgG has been shown to bind to Aβ deposits in brain in AD patients while a high IgG plaque labeling index was accompanied with reduced plaque burden suggesting that auto-Abs against Aβ may help to control Aβ burden [35]. Whether administration of IVIG, as a therapy, could further increase plaque labeling in patients with high IgG plaque labeling index or compensate reduced plaque labeling in patiens with low IgG plaque labeling index to further enhance the recognition and phagocytosis of Aβ deposits remains to be clarified. As presented here, we studied the penetration of peripherally administered exogenous IVIG into the brain and binding to Aβ deposits. By injecting human IgG into a mouse, we had the opportunity to visualize the distribution of IVIG in the mouse brain. We found that peripherally administered IVIG reached the brain parenchyma with sufficient concentrations to be detected, but that also significant regional differences existed. The highest concentration of human IgG was found in the septal hippocampus with a clear declining gradient toward the temporal end of this brain structure. Together with strong IgG immunoreactivity lining the ventricles, this finding indicates that the primary access route of IVIG to the mouse brain is through the choroid plexus.

To demonstrate the relevance of these findings *in vivo*, we found peripherally administered IVIG to bind to Aβ deposits in the brain parenchyma. Human IgG-bound Aβ deposits were accompanied by closely surrounding Iba-1 immunoreactive microglia. This suggests that IVIG not only can bind to soluble oligomeric Aβ species and potentially prevent Aβ fibrillization and toxicity, but IVIG can also bind to pre-formed Aβ deposits *in vivo *and possibly contribute to reduction of Aβ burden after initial Aβ deposition. As supported by our *ex vivo *data, surrounding microglia may also contribute to Aβ clearance after IVIG administration *in vivo*.

## Conclusions

The present data demonstrate that in addition to neuroprotective effects, IVIG promotes recognition and removal of natively formed human Aβ deposits by microglia. Our results suggest that natural Aβ Abs in IVIG interact with and promote the phagocytosis of Aβ deposits, resulting in enhanced microglia-mediated Aβ clearance. This has therapeutic relevance *in vivo *as we found that peripherally administered IVIG penetrates through the BBB and specifically binds to Aβ deposits in the brain parenchyma. These findings strongly support IVIG as a potential therapy to compensate the deprivation of naturally occurring Aβ Abs in AD that could alleviate the Aβ-induced toxicity.

## Competing interests

The Principal Investigators J. Koistinaho and H. Tanila have received funding from Baxter Innovations GmbH for their research projects in the University of Eastern Finland. All experiments and data analysis of this study were conducted in the University of Eastern Finland.

## Authors' contributions

JM participated in the phagocytosis assays and statistical analysis and drafted the manuscript. RP and SN carried out the phagocytosis assays and participated in the data analysis. KK performed toxicity studies, participated in the data analysis and helped to draft the manuscript. GG carried out fibrillization experiments and participated in the interpretation of the data. LP and TM carried out in vivo experiments and participated in the data analysis and interpretation of the data. WH and JG carried out immunohistochemistry, confocal microscopy and interpretation of the data. HT and JK participated in the design of the study and drafting and revising the manuscript. MK participated in the design of the study, data analysis and drafting and revising the manuscript. All authors read and approved the final manuscript.
